# Social support groups and policy recommendations for managing type 2 diabetes: perspective of healthcare professionals in Ghana

**DOI:** 10.3389/fcdhc.2025.1604828

**Published:** 2025-06-20

**Authors:** Christine Ahiale, Augustine Kumah

**Affiliations:** ^1^ Public Health Department, Ghana Health Service, Accra, Ghana; ^2^ Quality Department, The Bank Hospital, Accra, Ghana; ^3^ Department of Policy & Research, RIGHT Institute - Research on Interventions for Global Health Transformation, Accra, Ghana

**Keywords:** healthcare workers, social support groups, policy recommendations, type 2 diabetes, Ghana

## Abstract

**Background:**

Over the last decades, non-communicable diseases such as diabetes have remarkably increased due to rapid urbanization, unhealthy lifestyles, and ageing. The World Health Organization (WHO) indicates that the “diabetes epidemic” will continue in the coming decades, yielding enormous human and economic costs around the world. This study explores healthcare workers’ views on social support groups and policy recommendations for managing type 2 diabetes in Ghana.

**Method:**

This study utilized a facility-based cross-sectional qualitative research design to explore healthcare workers’ views on social support groups and policy recommendations for managing type 2 diabetes in Ghana between January and May 2023. Health workers who worked in the three selected regional hospitals for at least one year and at the diabetic clinics of the three selected regional hospitals of the respective regions at the time of this study were included. The study used purposive sampling to select 12 health workers (Nurses) as key informants. One (1) facility head of each facility and three (3) health workers, each working in the diabetic clinic of each regional hospital, were selected for key informant interviews. The interviews were recorded and transcribed in English. Notes from the interview were transcribed after the key informant interview. Data was imported into the Nvivo 7 software. The results were presented as prose, analyzed, and discussed in themes.

**Results:**

Major challenges facing people living with diabetes were the cost of medication, limited NHIS coverage, frequent morbidity and the chronic nature of the disease, putting psychological pressure on the patients. The policy recommendation was on mass education and the expansion of NHIS coverage.

**Conclusion:**

The study has noted some significant challenges faced in managing type 2 diabetes mellitus in Ghana. Addressing the diabetes epidemic in Ghana requires a comprehensive and multi-pronged approach, focusing on prevention, early detection, access to care, and diabetes education. Implementing the policy recommendations outlined in this study can significantly improve diabetes management in Ghana, reduce the burden of the disease, and enhance the overall health and well-being of the population.

## Introduction

Diabetes Mellitus (DM) is characterized by elevated blood sugar levels and is classified as a chronic metabolic disorder according to the World Health Organization (1). Diabetes is a leading cause of death in the world. As of the year 2019, about 463 million of the global population are living with diabetes, with projections further indicating that the number could rise by 51% to reach 700 million people by 2045 (2). About 24 million adults living with Diabetes Mellitus (PLWD) globally are found in sub-Saharan Africa (SSA), and this figure is predicted to rise to 55 million by 2045, an increase of 134%, with young people accounting for a disproportionate percentage of PLWD (2).

The global prevalence of type 2 DM is projected to increase to 7079 individuals per 100,000 by 2030, reflecting a continued rise across all regions of the world (3). The WHO ranked DM as the 9^th^ leading cause of death globally (1). Moreover, the hospitalization and mortality rates from type 2 DM are increasing across countries of all economic income. In Nigeria, for instance, the hospital admission rate for type 2 DM was estimated at 222.6 per 100,000 population, with hyperglycemic emergencies (4). The overall mortality rate was 30.2 per 100,000 population, with a case fatality rate of 22.0% (4).

In Ghana, numerous studies conducted on the prevalence of Type 2 Diabetes Mellitus (T2DM) in various geographical locations have consistently indicated a high prevalence (5–7). A study in the Central Belt of Ghana revealed a substantial increase in type 2 diabetes admission rates, rising from 2.36 per 1000 admissions in 1993 to 14.94 per 1000 admissions in 2014, representing a staggering 633% surge over the 31-year period (8). Over the same 31-year period, inpatient diabetes fatality rates witnessed an increase from 7.6 per 1000 deaths to 30 per 1000 deaths8. Additionally, a retrospective cross-sectional study conducted on predominant complications of type 2 DM in Ghana revealed that the prevalence of macrovascular and microvascular complications of type 2 DM was 31.8% and 35.3%, respectively (9). The study further revealed that the prevalence of neuropathy, nephropathy, retinopathy, sexual dysfunction, diabetic ketoacidosis, and hypoglycemia was 20.8%, 12.5%, 6.5%, 3.8%, 2.0%, and 0.8%, respectively (9). All these complications of type 2 DM can be minimized if much effort is directed toward improving the QoL of people with type 2 DM.

Ghana intends to prevent and control chronic non-communicable diseases (NCDs) by implementing the broad approach outlined in this national policy. Some of its primary sources of inspiration are the World Health Organization (WHO) and other national and international policy and strategy papers (10). Current definitions classify illnesses or conditions that affect people for a prolonged period and for which no known cause can be transferred from one affected individual to another as chronic non-communicable diseases (NCDs). The World Health Organization (WHO) lists the following conditions as non-communicable diseases (NCDs): mental illnesses, cancer, diabetes, chronic respiratory diseases, vision and hearing impairment, oral diseases, bone and joint problems, genetic disorders, and cardiovascular diseases, particularly heart disease and stroke (10). Chronic NCDs are responsible for 44% of premature deaths and 60% of the 58 million estimated deaths that occur globally each year (10). Age-standardized disability-adjusted life year (DALY) rates for non-communicable diseases (NCDs) are higher in low- and middle-income countries (LMICs) than in high-income countries (10). Eighty per cent of deaths from chronic diseases occur in LMICs, home to the majority of the world’s population. People in these countries usually get sicker at younger age, have more prolonged illnesses, and die sooner than people in high-income countries (10).

## The Buffer Theory of Social Support

**Figure 1:**
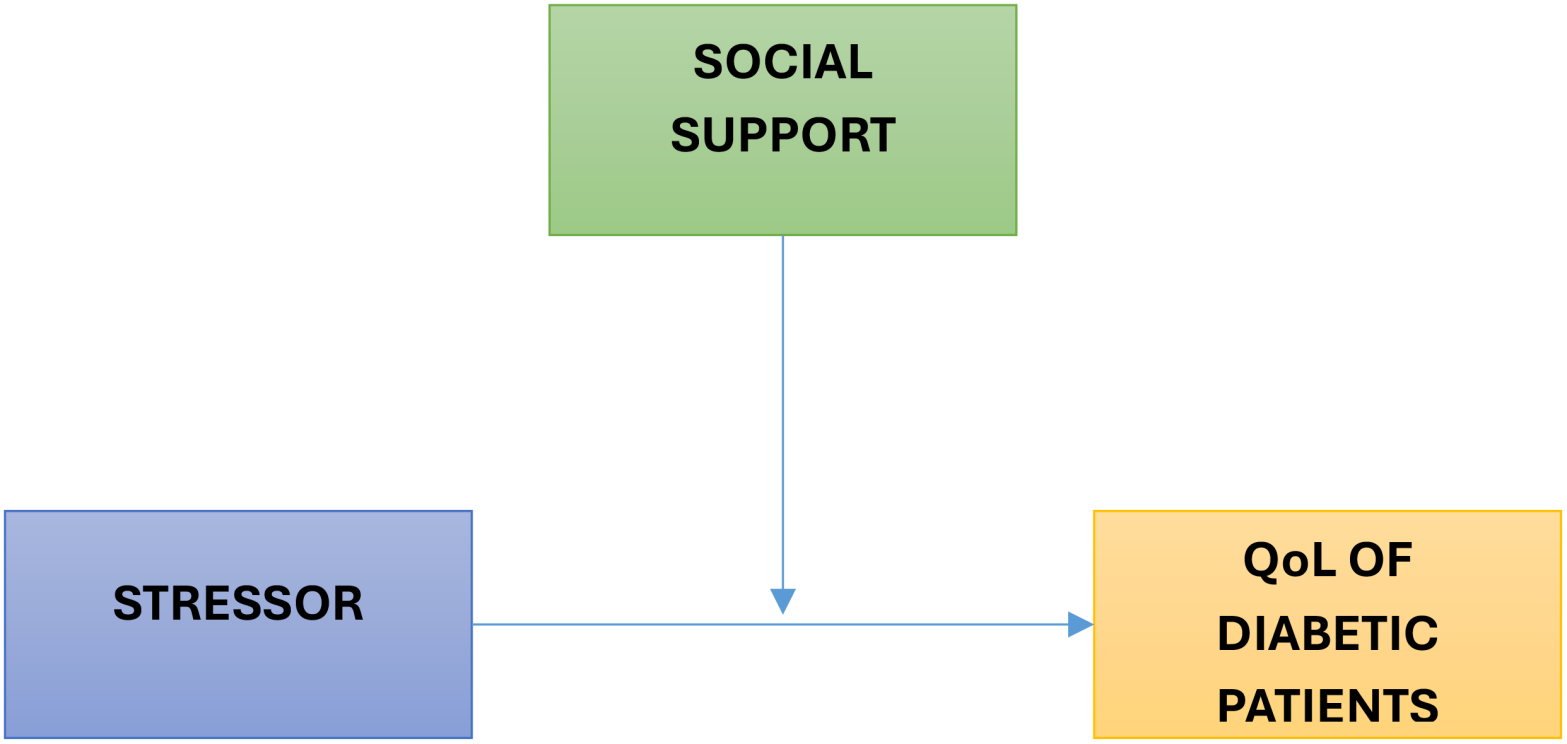
Buffer theory of social support.

Alloway and Bebbington introduced the Buffer Theory of Social Support in 2018. Later, the idea was developed by Nan Lin and his team (11). The idea is that social support functions as a buffer, lowering the assessment of a stressful experience and making it seem less dangerous. According to Lin et al. (2015), buffering is any social support mediating between stressors and health. According to the Buffer Theory of Social Support, social support networks or groups mitigate the impact of unfavorable stressors that cause illness and disease (11). According to the study, social support groups or networks may not directly affect an individual’s health outcome (11). Still, they can serve as a “buffer” to protect them from the negative impacts of their surroundings when they’re under stress. This shows that by reducing or preventing diabetic patients’ stress appraisal response, social support may mediate between the stressful experience and the stress reaction. The idea has demonstrated that social support generally affects people under stress, but the kind and amount of social support also matter greatly (**Figure 1**).

Social support is one of the emotion-oriented coping mechanisms with the potential to influence life quality (12). Studies have shown a significant relationship between health and social support, so people who receive higher social support have better health (2, 12, 13). Diabetes disturbs the patient’s daily performance and social activities, changes his/her capability for performing regular roles and responsibilities, and creates new roles for him/her (12, 13). The relationship with the spouse, children, parents, sister, brother, friends, and other social network members differs from before. These people depend on others and can support others to a lesser degree. Therefore, their interactions with others are limited, and they may be isolated in society. Thus, their need for social support increases. Social support affects the control of diabetes through two processes: a) the direct effect of social support via behaviors related to health, such as encouraging healthy behaviors, and b) the moderating effect of social support, which helps in the moderation of acute and chronic nervous pressure on health and increase of compatibility with the nervous pressure of the diabetes disease (2, 12, 13). This study explores healthcare workers’ views on social support groups and policy recommendations for managing type 2 diabetes in Ghana.

## Methods

### Study design

This study utilized a facility-based cross-sectional qualitative research design to explore healthcare workers’ views on social support groups and policy recommendations for managing type 2 diabetes in Ghana between January and May 2023.

### Study area

This study was carried out in 3 regional hospitals across the three ecological belts in Ghana: the Northern Region, the Ashanti Region, and the Greater Accra Region. These regions were purposefully selected because they are the most populated regions in the northern, middle, and southern belts, respectively, and have the highest burden of diabetes in Ghana.

### Study population

Health workers who worked in the three selected regional hospitals for at least one year and at the diabetic clinics of the three selected regional hospitals of the respective regions at the time of this study were included.

### Sampling method

The study used purposive sampling to select 12 health workers (Nurses) as key informants. One (1) facility head of each facility and three (3) health workers, each working in the diabetic clinic of each regional hospital, were selected for key informant interviews. The participants were selected in accordance with the inclusion criteria, which required at least one year of experience working at diabetic clinics in the selected regional hospitals. This sample size was deemed appropriate for a qualitative study of this nature, as it allowed for in-depth exploration of diverse perspectives across three major ecological zones in Ghana—Northern, Middle, and Southern belts. The composition of the sample, which included facility heads and frontline healthcare workers from each region, ensured both breadth and depth of information. The sample size was sufficient to reach data saturation, where no new themes or insights emerged during later interviews, thus reinforcing the credibility and rigor of the findings.

### Study material

Data was gathered through in-depth interviews with healthcare providers. The interviews aimed to explore their views on social support groups, challenges in diabetic management and policy recommendations for managing type 2 diabetes in Ghana. A structured interview guide was used to derive information from the participants.

### Data analysis

The qualitative data collected from the key informant interviews were transcribed and analyzed thematically using NVivo 7 software. The analysis followed a schematic coding technique that involved several structured steps to ensure rigor and transparency.

First, open coding was conducted by reading each transcript line-by-line to identify significant statements and recurring ideas. These statements were assigned initial codes, which were derived inductively to remain grounded in the participants’ narratives. Following this, axial coding was used to examine relationships among the codes, allowing the grouping of related codes into broader categories. Subsequently, selective coding was employed to develop overarching themes that captured the central phenomena related to healthcare workers’ views on social support groups and policy recommendations for managing type 2 diabetes. A comprehensive codebook was developed to ensure consistency, including code definitions, sample quotations, and criteria for inclusion.

The thematic structure was visually represented through a schematic model to show the hierarchy of themes, interconnections between categories, and feedback loops where applicable. To enhance the credibility and trustworthiness of the analysis, several triangulation strategies were employed. Investigator triangulation involved two researchers independently coding a sample of transcripts. Differences in interpretation were discussed and resolved through consensus meetings, which led to further refinement of the codebook and themes. Data triangulation was ensured by collecting data from healthcare professionals across three geographically diverse regions (Northern, Middle, and Southern Ghana) and from both frontline clinic staff and facility heads.

In addition, peer debriefing was undertaken with an experienced qualitative researcher external to the study, who reviewed and critiqued the preliminary themes and interpretations. Analytic memos and reflective journaling were used throughout the analysis process to document analytic decisions, track emerging insights, and minimize researcher bias. These combined approaches contributed to a rigorous and credible thematic analysis process that faithfully represents the perspectives of the study participants.

### Ethical considerations

Ethical clearance was obtained from the Ghana Health Service Ethics Review Committee and the University of Port Harcourt Research Ethics Review Board. Before the commencement of the study, permission was obtained from the regional health directorates and hospital administrators of all selected regions and hospitals, respectively. Informed consent was obtained from each participant before the interview, and the participants were allowed to withdraw from the study at any time without penalty.

## Results

This study explored healthcare providers’ views on social support groups and challenges in managing type 2 diabetic patients in Ghana. The qualitative data from the study is discussed below. They are grouped into significant themes for easy understanding and better appreciation of the research questions raised in the study from the beginning of the study. The responses are hereby indicated below;

### Role of social support groups in diabetic management

Social support groups’ role in improving diabetic management in Ghana was not left out. Participants believed that the relevance of social support has yet to be well harnessed in Ghana. Some joint statements from which the teams were generated were;

*“There should be public education on. TV, social media, and other media are about what diabetic patients should eat and do in other health routines.”*

*“OK, so our social support groups can help us improve diabetic management by running a mass campaign on our social media platforms. We can make use of Twitter and Facebook, and we can even use them on the radio and television. Yes, there can be campaigns. What’s the disease about? The fact that it is chronic, that it will be with you for a lifetime if you have it.”*

*“To prevent or manage diabetes in Ghana, the NGOs are also trying their best through public education and announcements, public education.”*

### Role of families in the management of diabetes

The role that families of diabetic patients can play in improving the QoL of diabetic patients in Ghana was well appreciated by participants. Key participants’ responses are as follows;

*“I think they have to help them financially. And they should support them sometimes if they visit the hospital; they should come with them for checkups and check their health regularly.”*

*“Families can do a lot in helping to improve the health status of their relatives if they have this disease with them, especially when it comes to providing funds, and sometimes, to clients, who mostly forget their review.”*

### Role of government in diabetic management

On the issue of what the Government can do to improve diabetic care in Ghana, it was indicated that the government has much to do. Participants indicated that;

*“Reduction of some of these costs is necessary because it means that some of these patients have to come to their hospital at least once every month, or more than once every month. Some of them are hospitalized for days, and at the end of the day, their insurance doesn’t really cover many things, so the government needs to help these patients.”*

*“Social support groups can help us improve diabetic management by running a mass campaign on our social media platforms, using Twitter, Facebook, and even radio and television. Yes, there can be campaigns on what the disease is about. The fact that it is chronic will be with you for a lifetime if you have it, and the government can provide nutritional centers to help educate patients.”*

*“The government should motivate staff; it will help. I think the motivation is not there. Also, the clients should have something that will be given to them to motivate them to come to the clinic, especially those who usually come on time and who take their medication well, will be motivated to bring others to us or to help. It will encourage others also to take their medication. And more importantly, running education on radio and TV on the disease, bringing down medication prices.”*

### Role of Ghana Health Service/Health facilities in diabetic care in Ghana

Regarding the actions that major health service providers, such as GHS Health facilities, can take to enhance the management of diabetes in Ghana, participants emphasized that:

*With medication availability, we know that if some of these medications are given at the facility level, the prices are relatively lower than when purchased from outside pharmacies.*

*Everything is expensive now. Medications are expensive. In the laboratory, everything is expensive. Health insurance is doing little, so most of these patients don’t come to the hospital to be taken care of.*

*“Health service facilities can improve diabetic management in Ghana by creating awareness. One, I think awareness creation is key; when people are aware of the disease, they are halfway through how to protect and manage themselves or prevent themselves from getting it”*

*“For Ghana Health Service, they already have the policies there, but the implementation is a problem; they should get down to the grassroots and implement them.”*

### Challenges in Type 2 Diabetes Management in Ghana

The study revealed a diversity of challenges observed by participants in providing services for diabetes patients across the regions in Ghana. Here are some of the prominent perspectives shared by respondents:

*“When they are readmitted, they feel like they are doing all they were told. Do they take their medications? They follow the strict diet plan that has been given to them and all that. But at the end of the day, nothing seems to improve for them” (Participant 5)*

*“I’ll say challenges concerning diabetic patients in Ghana. I’ll say I feel like diabetic patients in Ghana need more counselling and advice compared to HIV patients. I think their awareness should go as it goes for the HIV patients” (Participant 3)*

*“The combination of the herbal and then the Orthodox treatments, because of our culture and tradition, they tend to. Add the herbal medicine to the Orthodox medicine or even neglect their orthodox medicines for herbal ones” (Participant 1)*

*“Ohh, currently, I’ll say yes. I’ll say yes because most diabetic patients are concerned about the disease being chronic. That is going to be with them for a fair number of years or an unknown number of years, and they are scared, as well as the fact that their medication is expensive and is not covered by NHIS.”*

### What interventions have been implemented so far to address these challenges?

Respondents mentioned different but effective interventions concerning the issues of interventions implemented to address the challenges. Participants indicated that;

*“Little has been done concerning because. Umm. Sometimes even the follow-ups become a problem because of a lack of resources, and no one is funding some of these things’ follow-ups on our patients who are diabetic and need to be constantly reminded of their medication.” (Participant 4)*

*“I feel that we are not putting much effort into the diabetic clinic because some patients feel they are just coming for their medications. But most of the time when they go to their hospitals, they have to go through the queue and all that as though they are new clients, who are now coming to see the doctors and so it becomes a problem for them to leave their work and then just come for a refill of medication when it’s going to be so tedious for them” (Participant 2)*

*“I think if we do health education, it will go a long way to help us both in the facility and the Community as a whole. We can form support groups or even form groups with those with similar conditions” (Participant 3)*

### Policy recommendations to improve the management of type 2 diabetes in Ghana

#### Availability of policy

Regarding whether there is any policy that addresses issues regarding diabetic management, facility officers were divided as to whether a policy was available or not. Those who asserted there is a policy noted that;

*“Yes, there is a policy by the Ghana Health Service. I think the policy is to ensure that the burden of NCDs is reduced to the barest minimum. So that it will render public health important.”*

*“Yes, and there are a lot of protocols and documents that they have brought to us to read and go through as to how to manage most of the cases we get in our facilities here.”*

*In the standard treatment guide, you know how to care for a people living with diabetes with high blood sugar. They give a protocol for that, and then those with very low sugar or those with ketoacidosis.*

#### Policy recommendations

Participants were also asked to make suggestions for policy recommendations and directions that will help tackle issues regarding diabetic management in Ghana. From the themes that were gathered from the study, Participants indicated that;

*“I think that mass publicity will do too. Eradicate some of these myths so that people will comply with their medications, and they know that once you have diabetes, it is not the end of the world for you.”*

*“I think that the diabetic drugs should be easily accessible to the patients, and then the prices should be slightly reduced. And I think nutritional centers should also be accessible so that patients can easily go there to check on their diets and be educated on things that they have to eat.”*

*So first of all, I will talk about diabetic drugs as they should be accessible, like easily accessible for patients to purchase these, if not for free. The charges can be subsidized for patients to purchase, and secondly, I’ll talk about providing nutrition centers or clinics at all our various facilities in Ghana. We lack nutritional centers. So, providing nutrition centers at our health facilities can help people visit there regularly for advice on their diet management.*

## Discussion

### Role of social support groups in diabetic management in Ghana

The study found that the role of social support groups is necessary for education, psychological support, and improving the quality of life of people living with diabetes by sharing ideas, especially on dietary regimens and self-care. The social support groups provide immersive support, providing educational information on the chronic nature of the disease to prepare people with diabetes to embrace their status and live healthy lives. This finding is supported by a study done by Carla K. Miller and Melissa S. Davis on the influential role of social support in diabetes management, which identified four dimensions of support; they provide appraisal support in choosing the appropriateness of food, informational support where factors affecting glycemic control are identified, emotional support which encourage them (14). Social support is believed to be an essential component of effective diabetes management. Due to the nature of the disease, people living with diabetes need psychological support so that they do not feel alone and give up on their treatment.

Diabetes is a chronic condition that affects millions of individuals worldwide, with significant physical, emotional, and psychological implications. Managing diabetes effectively requires more than medical intervention; it often necessitates a holistic approach that includes lifestyle changes, emotional support, and education (13, 14). Social support groups have emerged as an essential component of diabetes management, offering individuals a platform to share experiences, gain knowledge, and receive emotional assistance. Social support groups provide an invaluable forum for individuals with diabetes to connect with peers facing similar challenges. These groups offer a safe space where members can openly discuss their experiences, share coping strategies, and exchange information on diabetes management. Research has shown that peer support enhances diabetes self-management (15). Through interaction with others who have faced similar situations, individuals gain practical insights into managing their condition effectively.

The emotional impact of living with diabetes should not be underestimated. Diabetes often leads to stress, anxiety, and even depression. Social support groups offer emotional support, reducing feelings of isolation and depression among participants (16). Regular meetings and interactions with supportive peers can significantly improve an individual’s psychological well-being, ultimately contributing to better diabetes management (16).

Effective diabetes management requires a solid understanding of the condition and its treatment options. Social support groups often invite healthcare professionals, nutritionists, and diabetes educators to provide information and resources. This educational aspect empowers individuals with knowledge, enabling them to make informed decisions about their diabetes management (17). Moreover, the group setting encourages participants to participate actively in their care, leading to better self-advocacy and self-efficacy.

Social support groups create a sense of accountability among their members. Regular meetings and interactions with peers can motivate individuals to adhere to treatment plans, maintain a healthy lifestyle, and diligently monitor their blood sugar levels (18). Knowing that others share their goals and challenges can motivate individuals to stay on track with their diabetes management. Effective diabetes management is not only beneficial for individuals but also for healthcare systems (18). Research has demonstrated that individuals actively engaging in social support groups are likelier to achieve improved glycemic control and lower the risk of diabetes-related complications (19). This, subsequently, can result in decreased healthcare costs linked to diabetes management.

Social support groups play a pivotal role in diabetes management by providing peer support, enhancing psychological well-being, offering education, promoting accountability, and potentially reducing healthcare costs. Engaging in these groups can significantly improve an individual’s ability to cope with diabetes and lead a healthier, more fulfilling life. Healthcare providers and policymakers should recognize the value of social support groups and incorporate them into diabetes care programs to optimize patient outcomes and quality of life.

### Role of families in the management of diabetes in Ghana

This study found that families of people living with diabetic can help improve their quality of life. Most respondents mentioned that family support is necessary in diverse ways: emotionally, through diet and medication management, and financially. Diabetes is a chronic condition that not only affects individuals but also has a significant impact on their families. Family members’ role in managing diabetes is crucial for achieving better health outcomes and improved quality of life for those with the condition.

Living with diabetes can be emotionally challenging. Family members can provide emotional support by offering a listening ear, empathy, and encouragement. Studies have shown that emotional support from family members can reduce stress and improve psychological well-being for individuals with diabetes (20, 21). Family members can contribute to diabetes management by adopting healthier eating habits as a family. They can help plan and prepare balanced meals that align with the individual’s dietary requirements. Involving the family in meal planning ensures a supportive environment that promotes healthy eating (22). Encouraging physical activity is vital for diabetes management. Family members can participate in regular physical activities with the individual, making exercise more enjoyable and motivating. Family physical activity can help control blood sugar levels and improve overall health (21, 23).

Family members can assist in medication management by reminding the individual to take their medications as prescribed and helping them keep track of medication schedules. This can contribute to better adherence to treatment plans (24). Family members can assist in monitoring blood sugar levels, especially in cases where the individual has difficulty doing it themselves. This helps in the timely detection of fluctuations and allows for appropriate adjustments in treatment plans (19). Family members can become knowledgeable about diabetes by attending educational sessions with the individual. This enables them to effectively advocate for the individual’s healthcare needs, especially in healthcare settings (20, 21).

Family members can play a role in reducing the risk factors associated with diabetes. This includes supporting smoking cessation efforts, promoting stress management techniques, and helping to maintain a healthy weight as a family (25).

### Challenges in type 2 diabetes management in Ghana

#### Non-adherence to dietary regimen

People living with diabetes major worries are about their dietary regimen, where they are made to follow a specific diet plan. People living with diabetes face various challenges in adhering to dietary restrictions. This includes resisting the temptation of high-sugar and high-carb foods, dealing with cravings, and navigating social situations where unhealthy foods may be prevalent. Emotional challenges are also common, with many individuals experiencing guilt when they deviate from their dietary plans and frustration when their blood sugar levels do not respond as expected despite their efforts (24, 26, 27). Diet restrictions go beyond the physical aspect of food choices; they encompass a significant social and emotional impact. Patients may feel isolated or left out during social gatherings, leading to feelings of loneliness and alienation. The emotional toll includes guilt, anxiety, and stress associated with managing dietary restrictions. These emotions can affect the overall well-being of individuals living with diabetes (26).

Despite the challenges, there are coping mechanisms and strategies that individuals adopt to manage their diabetes diet restrictions effectively. These strategies may include meticulous meal planning, preparing healthy snacks, and adhering to portion control. Communication plays a vital role, as participants found that sharing their dietary needs with friends and family often led to increased support and understanding, mitigating the social challenges. Effective management of diet restrictions can lead to positive health outcomes, such as improved blood sugar control and reduced risk of complications (28). Weight loss is another potential benefit, but emphasizing overall well-being, including psychological health, is essential and equally significant in diabetes management (27).

#### Prolong treatment (lifetime medication) and adherence

Maintaining consistency in taking prescribed medications, often multiple times a day, can be challenging. Patients may encounter obstacles that affect their ability to adhere to the regimen, such as forgetfulness, medication side effects, or complex dosing schedules (29). Taking medication for a lifetime can significantly influence an individual’s daily life. Patients must incorporate medication routines into their daily schedules, potentially affecting work, social activities, and overall quality of life. Balancing medication with other aspects of life can be demanding (19, 29). People living with diabetes may experience frustration, anxiety, or even depression related to the ongoing management of their condition. The constant reminder of their illness can affect their mental well-being (19, 27, 29).

Accessibility and affordability of diabetes medications are critical concerns. Some patients may need more insurance coverage to avoid financial challenges, especially with inadequate insurance coverage. These concerns can lead to non-adherence or suboptimal treatment. The long-term implications of medication adherence are significant. Failure to manage diabetes effectively through medication can result in serious health complications, such as cardiovascular disease, kidney problems, and nerve damage (25, 30, 31). Patients may require ongoing support and education to navigate the challenges of lifelong medication. Healthcare providers, diabetes educators, and support networks are crucial in helping patients understand their medication regimens, cope with emotional challenges, and address any barriers to adherence (30). Despite the challenges, individuals with diabetes can learn to self-manage their condition effectively. Empowerment through education, self-monitoring, and lifestyle changes can enhance their ability to adhere to medication and maintain better control over their diabetes (27).

Adhering to a daily medication regimen for an extended period can be demanding. Missing doses or being inconsistent with medication timing can lead to fluctuations in blood sugar levels and hinder effective diabetes management (28). Some individuals with diabetes require multiple medications, including insulin, oral drugs, and other injectables. Managing multiple medications with varying dosages and administration methods can be overwhelming and increase the risk of medication errors (30). Diabetes medications can have side effects, such as weight gain, gastrointestinal issues, or hypoglycemia (28). Coping with these side effects while striving for medication adherence can be emotionally and physically challenging (19).

The cost of diabetes medications can be a significant barrier to adherence, especially for those with limited or no insurance coverage. High medication costs may lead to rationing or skipping doses, potentially compromising health outcomes (31). Understanding the purpose of each medication, its potential side effects and the importance of adherence can be challenging for some patients. Limited health literacy can impede effective self-management (27, 32).

#### Stigma and social discrimination

Taking medication in public or disclosing one’s diabetes status to others can lead to feelings of stigma or self-consciousness. This may impact social interactions and the willingness to adhere to medication in various settings (33, 34). The fear of diabetes-related complications, such as cardiovascular disease, neuropathy, and vision problems, can be a constant source of anxiety for individuals with diabetes. This fear can influence motivation for medication adherence (20, 33, 34). A robust support system, including healthcare providers, diabetes educators, and peer support groups, is vital for addressing these challenges. Support can provide guidance, emotional support, and motivation to continue medication management (20). These challenges encompass emotional, financial, and social aspects and necessitate a comprehensive approach to diabetes care.

#### Cost of treatment

Another challenge that diabetes patients face is the cost and availability of medication. The respondents point to the fact that it is difficult for the patients to afford their medications, especially if they must buy them from pharmaceutical shops other than the health facility. This finding can be resonant with a review on why diabetes medication is so expensive and what can be done to control its cost, which found that the cost of antihyperglycemic medicines, especially insulin, has become a barrier to diabetes treatment (35–37). These studies recommended a policy intervention to control the cost of antihyperglycemic medication (35, 36). Respondents suggested that the Ghana National Health Insurance Scheme should cover diabetes medication, and not only be covered by the National Health Insurance, but also ensure its availability at the various health facilities.

When the medications are expensive and unavailable at the health facility, it increases the probability of patients defaulting on treatment. A dominant factor causing complications among people living with diabetes in Ghana is the use of herbal medicine, where they believe that with the herbal medicine, they can be cured of the disease. The high cost and limited availability of medications at the health facilities compel some of these patients to resort to the use of herbal medicines, which later give them more complications. A significant proportion of people living with type 2 diabetes are aged above 50 and often fall into the category of either being unemployed or retired from active service. This demographic can face challenges in affording their medications. Generally, medications are more accessible and affordable at health facilities. Hence, it is crucial to emphasize the availability of antihyperglycemic medications at these facilities. This ensures better affordability, helps prevent complications resulting from non-adherence, and reduces the rate of treatment default.

### Policy recommendations for improved diabetic management in Ghana

#### Public health awareness and education

On policy recommendation, most health care workers advocate for a policy to increase health promotion for the disease as done in other communicable diseases such as HIV. Media houses can partner with the government to educate the public on the chronic nature of the disease, adherence to treatment and dietary regimens. Implementing policies that support diabetes education and self-management empowers individuals to participate actively in their care. When individuals are well-informed about their condition and equipped with the necessary tools for effective management, they achieve better health outcomes and experience an enhanced quality of life (22).

To combat diabetes effectively, Ghana should prioritize raising awareness about the condition and providing comprehensive diabetes education programs. This includes initiatives in schools, communities, and healthcare settings to educate the public about diabetes risk factors, symptoms, prevention, and self-management (38). Culturally tailored educational materials and campaigns can enhance the impact of these efforts. Ghana should establish a systematic and widespread screening program for diabetes, mainly targeting high-risk populations. This includes routine blood glucose testing during healthcare visits and community-based screening programs to identify individuals with undiagnosed or prediabetes (4). Timely detection is crucial for early intervention and improved outcomes.

#### Establishment of diabetic nutrition clinics and centers

Another innovative suggestion is establishing nutritional centers that can be easily accessible to diabetes patients. The Ghana health service can enforce diabetes clinics specifically for diabetes. At the diabetes clinics, the patients should not be queued with other outpatients. Doing this causes many delays, which discourages people living with diabetes from frequent reviews. The cost of medication must be considered in policy to ensure medications are subsidized or made accessible, as in HIV and TB medications. The continual availability of anti-glycemic medicines at health facilities is the surest way to ensure compliance with the diabetes treatment regimen. Diabetes is a growing health concern in Ghana, with significant implications for public health and the overall well-being of its citizens (37). Effective diabetes management, as suggested by many other studies, requires a multifaceted approach, including policies that promote prevention, early detection, treatment, and education.

#### Complete coverage of diabetic treatment under the NHIS

Policymakers should prioritize improving access to diabetes care, including affordable medications and supplies. This can be achieved by expanding the coverage of health insurance programs to include diabetes-related services and medications. Reducing the financial burden of diabetes management is essential for ensuring that individuals can access and afford the necessary treatments (39). Diabetes is associated with significant healthcare costs. Effective policies that support diabetes management can help reduce these costs in the long term. This includes policies to prevent complications through better glycemic control and promoting cost-effective interventions (40). Lowering healthcare costs benefits both individuals and healthcare systems. Policies that improve access to diabetes care directly impact treatment outcomes. When individuals have access to affordable medications, regular check-ups, and diabetes education, they are more likely to manage their condition effectively (39). Accessible care also reduces the risk of diabetes-related complications.

#### Lifestyle regulation and modification for diabetic patients

Ghana should implement policies that encourage healthy lifestyles, including nutrition and physical activity. This includes initiatives to reduce the consumption of unhealthy foods and promote physical activity in schools, workplaces, and communities (41). Additionally, policies could support the development of safe and accessible spaces for physical activity. For example, policies encouraging lifestyle modifications and regular screening can help identify individuals with prediabetes or undiagnosed diabetes, allowing timely intervention (42). Policymakers should promote diabetes self-management by providing resources and support for individuals with diabetes. This includes diabetes education programs, peer support groups, and telehealth services to help individuals monitor their condition and adhere to treatment plans (7).

#### National diabetes registry

Creating a national diabetes registry can facilitate better data collection and analysis. This information can guide policy development and resource allocation for diabetes prevention and management (4). A registry can also help identify trends and disparities in diabetes prevalence and outcomes. Ghana should promote collaboration among healthcare providers, government agencies, non-governmental organizations (NGOs), and community organizations to establish a well-coordinated approach to diabetes management. Partnerships can result in more efficient implementation of diabetes policies and programs (39).

#### Diabetic research funding

Continuous research and evaluation of diabetes policies and programs are essential. Policymakers should allocate resources for ongoing monitoring and assessment to ensure policies achieve their intended outcomes and make necessary adjustments (7, 43). Policymakers can stimulate research and innovation in diabetes management. Policies that allocate resources for diabetes research, support clinical trials, and incentivize the development of treatments and technologies can lead to advancements in diabetes care (31, 44, 45).

### Limitation

This study is limited to only three health facilities in three selected regions out of the 16 regions in Ghana. This means that the study findings might only apply to some regions in Ghana. Secondly, the study adopted a cross-sectional design, restricting our ability to establish causation or track changes over time.

## Conclusions

The study has noted some major challenges faced in managing type 2 diabetes mellitus in Ghana. Addressing the diabetes epidemic in Ghana requires a comprehensive and multi-pronged approach, focusing on prevention, early detection, access to care, and diabetes education. Implementing the policy recommendations outlined above can significantly improve diabetes management in Ghana, reduce the burden of the disease, and enhance the overall health and well-being of the population.

## Data Availability

The original contributions presented in the study are included in the article/supplementary material. Further inquiries can be directed to the corresponding author.
